# Editorial Department

**Published:** 1882-10

**Authors:** 


### The Bistoury.

ELMIRA, N. Y., OCTOBER, 1882.

The BISTOURY is published Quarterly, upon the first of April, July, October and January, at Fifty Cents a year in advance.

### Editorial Department.

REWARDS FOR SCIENTIFIC INVESTIGATIONS.—The French Academy of Science has published a list of prizes offered for investigations into the causes and cure of various diseases. The prizes are large, and in cash. This is a stimulant for scientific investigation that has never been imitated in this country. We have hosts of careful, patient, competent scientists who have the appliances and the desire to make just such investigations, but are debarred therefrom for lack of pecuniary means to support themselves while thus employed. If some of our wealthy universities or societies would imitate the French Academy in offering sufficient prizes for the discovery of the causes which are instrumental in developing certain diseases, much good to our race would accrue therefrom.

---

SURGERY EXTRAORDINARY.—We saw a statement, in one of the Philadelphia newspapers, to the effect that a chap who had suffered a railroad injury, was conveyed to one of the many excellent hospitals of that city, for repairs. Meeting an acquaintance, months afterward, he was asked whether he had been kindly cared for during his stay in the hospital. He replied about as follows: "O, I have no reason to complain; for they amputated both my feet, removed one of my clavicles, cut off my right arm, resected my left elbow, trephined my skull, took out a piece of my inferior maxillary, sawed my left os-innominatum in two, and were about to exsect a rib or two, when a fire occurred in the establishment, and a policeman got away with the remainder of me—and here I am."

---

MULTIPLICATION BY "FISSION."—Certain infusoria multiply their kind by a process known as "fission," in which the animal constricts itself in the center of its body until it is literally cut in two, when each separate piece becomes a perfect animal, swims away, repeating the process in a few hours. In this manner, thousands of creatures, known to the microscopist as *paramecium*, are produced in a few hours.

Now, since this is one of the first methods of reproduction, it does not become quite so difficult to understand that Adam and Eve story, in which it is related that Adam fell asleep, when a rib was taken from his side, from which his companion Eve, was manufactured. Doubtless, the historian was not "up" in natural history—particularly the microscopical portion of it—and took the act of "fission" to be a surgical operation for the resection of a rib. It would seem more than probable that Adam did precisely what we see the *paramecium* doing daily, to wit., he constricted himself until he was cut in two, when Adam retained his personality in one-half of the division, while the "other half" became gentle Eve. And, plainly enough, from this very circumstance, has come down to us through the ages, that term by which one's wife is generally known—his "better-half."

---

AUTUMN DAYS.—Now the autumn days have come; the frost is fast turning the leaves into the brilliant hues which characterize the season. The hillsides are varied in the contrast between the new sprouting wheat and the brilliant reds and browns of the buckwheat stubble and shocks.

The purples and yellows of wild flowers are not yet lost. Nature is always beautiful, but at this season she seems to put on her most attractive dress, when vines and trees are loaded with fruit, cornfields picturesque in their rows of miniature tents, the husks protruding at the sides, telling of a crop which a few months ago was almost despaired of, while the tobacco sheds, show through their open shutters the success which has crowned the labors in that direction.

People of Elmira, to your horses, your wagons, and, if you have neither, foot it into the country around, and see what nature has been doing for you. This is a beautiful world, and it needs only to cultivate your tastes to be able to enjoy a life which now may seem a burden. Go up into the hills. See how Nature has provided for those who neither "reap nor spin"—nuts and mass in abundance, with berries and wild grapes for the birds.

Gather your lesson in your rides and walks; let your ideas expand beyond self and selfish surroundings; and come home happier, and thankful that your "lines have fallen in pleasant places."

---

OUR CONTRIBUTORS.—We heartily welcome another able and permanent contributor to the BISTOURY in Dr. Blackham, ex-President of the American Society of Microscopists. His second article appears in the present number, showing what may be expected of him in the future. With such able writers as Ben. H. Pratt, Ausburn Towner, James B. Chandler, and Dr. Blackham for permanent contributors, our subscribers will see that they are likely to get the full value of their subscription fee.

The contributions are of unusual interest this quarter, and will repay you for a careful reading of them.

To our medical readers—by far the largest in number of our regular subscribers—we would say that we give great care and attention to the preparation of the "Medical Items and News" department, reading and culling from all the important medical literature of the country the transpirings in the medical world. In the short, crisp articles (the main points and facts of much larger ones) are given you a condensation of the important medical news.

The BISTOURY enjoys by far the largest circulation of any medical journal published in the United States. We have subscribers in every State and Territory. It is taken largely by the country practitioner of medicine, who can illy afford the larger and more costly publications. For fifty cents a year, we give condensations of all that is written in our profession, worthy of consideration. The BISTOURY runs itself. No effort is made to increase its circulation or to secure advertising patronage. Its own merit sustains it, constantly increasing its patronage.

---

LATIN IN SCHOOL.—The poet calls attention to the fact that the sweetness of the rose would not be changed, even if called by another name. Yet, nevertheless, if one child was taught to call it a turnip, and another a cabbage, there would arise a slight difficulty in procuring the flower, where it was known under its proper name.

Now, it seems to have become customary, of late years, for persons engaged in compiling school books and teaching English and other languages, to adopt some method of their own as to the nomenclature of parts of speech or the pronunciation of words. Our attention was directed to this, the other day, by an application from one of the boys for an explanation of his latin lesson. We found that what in our school-boy days had been impressed upon our memory, and in some instances hammered into us *vi et armis*, under the manipulation of modern authors and school masters had been tortured into "error." As for the pronunciation of the latin language, the contention between two colleges has given a quasi authority for two methods, and there seems to be no disposition to endeavor to settle the dispute, either in favor of euphony or harshness. We were taught the soft pronunciation, which is approached by the Italians, and is most generally used in the Roman Catholic church, an authority which might, at least, be accepted for its own language. Yet, we would gladly learn of a settlement of the question on either side, so that our children might be taught a language as accepted by all, and not half the world.

On the other questions involved in this idea, we may say more at a future time, but just now, time is up and space exhausted.

---

POLITICAL.—The BISTOURY has nothing in its mission which would lead it to treat of politics, except only so far as the questions of the day bear upon the general good of mankind without regard to party organizations.

There seems, however, to have awakened in the minds of the citizens of this country a realization of the frightful depravity into which the politics of the nation has drifted, and with that knowledge we are glad to note arising a determination to elect to office only such men as are fitted for the place. Bossism and the party lash have lost their power, and enough good citizens are now to be found willing to vote only such a ticket as their knowledge of the men on it warrants them in endeavoring to place in the offices.

Men will no longer go to the polls and carelessly vote for parties for offices of responsibility whom they would not trust in their own establishments.

We have no mission here to advocate the claims of any political party, no matter what our own proclivities may be. Nor have we any desire to call attention to the merits or demerits of any party. The point we wish to ask our readers attention to, is that they will realize exactly what is the nature of the offices we are called upon to select men for. We hear daily this office or that one spoken of as "a nice thing to have," "a fat sinecure," &c., &c., and the character of public office seems lost sight of.

People do not realize that government, local, state or national is only a certain necessary business duty for which the public employ clerks, and these clerks are designated by certain titles, as President, Governor, Sheriff, Tide Waiter, etc.—all servants; and servants which ought to be selected with the same careful study for honesty and fitness as we would use in employing help in our own private business affairs.

These men are selected, or ought to be, to discharge certain duties which belong to their offices and which are necessary to the public weal. Whether of late years the re-election of themselves and others to office has not been their main business, those who have watched public affairs will determine.

We welcome cordially the growing public feeling of setting these things to rights, and feel assured that its benefits will be felt in not only a better administration of our affairs, but also in the increasing respect of all nations for Republican institutions.

---

MICROSCOPIC GERMS.—After reading Dr. Blackham's article, in this number, upon communicable diseases, and the address of Dr. Kammerer, Imperial Health Officer to the city of Vienna, one is impressed with the remark recently made in our hearing that, "it is getting to be dangerous to *try* to live!"

When it is known how we are surrounded with microscopic germs and parasites, that find lodgment upon our persons, are breathed into our lungs, and mingled with our food, the wonder is that we escape sickness as well as we do. Dr. Kammerer, above referred to, states that the milk of diseased cows—as well as their meat—contain tubercles, which, when taken into the stomach, as food, multiply in the system of the child as well as the adult, and give rise to tuberculosis or consumption. He further states his belief that infection by this channel, is as fruitful a source for consumption among the young, as from hereditary taint.

The house-fly carries upon his proboscis certain baccilli, obtained from its putrefactive food, that find their way into our circulation, while he is drinking moisture from our lips. The water we drink not only contains germs that prostrate us with typhoid and other fevers, that endanger life, but contains tubercles, and eggs of the tape-worm, as well. Pork endangers us to *trichina spiralis.* Fruits are the receptacles of the eggs of insects that hatch in our stomachs and intestines, to have their young give rise to inflammations that may produce death. And so, on every hand, we are confronted wlth these infinitesimal creatures which, by their enormous reproductiveness, block up the channels of our circulation and prostrate us on beds of sickness.

To strap a microscope upon one's back when he goes to market, that he may be prepared to inspect his contemplated food for parasites or their eggs, would be attended with more trouble than any timid man would be willing to subject himself to. So, we must content ourselves "to go it blind," and trust to thorough cooking and honest butchers that will not sell us the meat of sickly cattle.

---

MEDICAL SOCIETIES.—There exists a clause in the code of ethics that forbids the physician permitting his name to appear, in the newspapers, in connection with any surgical operation, or with any case that may be under his care. All sorts of devices have been employed to "keep within the letter of the law," yet secure the advantages of the "advertising" that such notices of the press may afford. One of the methods employed by the physician anxious for advertised honors is to see that his paper, read before the medical society, receives proper prominence in the newspaper report of the "minutes of the meeting." He is anxious that the dear public should be apprised of the unusual success that has attended his peculiar treatment for diphtheria, in which one hundred per cent. of his patients made a rapid recovery. Another reports the marvelous cures of ailments of the female pelvic organs; the better treatment of which is permitted by means of his new-fangled, double, bivalved, self-retaining, illuminating speculum. Still another details, in scientific phraseology, how he extirpated a lardaceous encephaloid from Scarpa's triangle, or an adeno-sarcoma from beneath the sterno-cleido-mastoides, that was characterized by the formation of a profundity of glandular acini. Of course, the average reader is either mystified as to whether Scarpa's triangle refers to a base ball ground, or whether the essayist is really as great a surgeon as the handling of scientific words would indicate. Of the final decision of the reader, the doctor must remain in ignorance, being content with the knowledge that his name appeared in connection with a case, so bringing it prominently before the good people whose patronage he seeks—and he being perhaps, worthy of it. We do not intimate that this method of advertising is reprehensible or commendable. We only point out a very common way of overcoming what would seem to be, to very many physicians, an obnoxious rule of ethics, to elude which they resort to a plan that is quite as opposed to the spirit of that rule, as a more open means of advertising through a "notice of an operation performed," in the daily papers.

---

A MORGAN MONUMENT.—These be indeed monumental days. What with statues erected to the memory of soldiers, statesmen and the prospective one to the sire of us all—Adam, the country is being beautified with artistic designs to commemorate the triumphs and achievements of men. And now, as though the eminent dead had all been suitably remembered in the granite and bronze mementoes, and money is running riot in search of somebody to whom a monument should be erected, a lot of stupids, in Batavia, have absolutely expended $2,500 in a bronze statue of one Wm. Morgan, whom history suitably describes as a "drunken wife-beater and thief." It seems that "A National Christian Association"—whatever that may be—interested themselves in this peculiar enterprise that the meritorious achievements of this fellow, in the wife-beating accomplishment, might be suitably enshrined in the hearts of the people, in tablets of granite and bronze. Of course, sensible people have long since discharged from their minds any idea that Morgan was in any sense a martyr to Masonry. Masonry did not then, nor does it now, hang by so slender a thread as to be severed by one insignificant human being. That portion of the tablet, therefore, that refers to him as having been "murdered by Free Masons, for revealing the secrets of their Order," is as false as it is shameful, and a disgrace to the displayers of the libelous words. Now, let this "National Christian Association" erect another monument in the same square with Morgan's. Let the figure be in bronze, of heroic size, of a primeval man, wearing a silk hat and pair of spurs, tightly holding a money bag in both hands. Let the tablet bear the following inscription:

JUDAS ISCARIOT.<br>A Character in Ancient History,<br>who took<br>30 Pieces of Silver—<br>All He Could Get!<br>For one little transaction on the street.

Erected by the "National Christian Association," to commemorate a principle—"Take all you can get and be content."

---

A BAD ELEMENT.—As an evidence of the existence of a bad element in society—one that endangers the capitalist and working man, alike, might be cited a conversation had in our hearing but a few days since. The theme related to the purchase of real estate in Elmira, and the conclusions drawn might apply as well to most towns and cities. One of the party had invested in real estate in a distant city, upon which he was interrogated somewhat as follows:

"Why don't you purchase property in Elmira? Would not the investment be quite as good as elsewhere?"

The reply was: "No. Property is depreciating in Elmira, instead of advancing." "Why so?" "Because of bad influences at work which destroy confidence." In explanation of this statement, after the citation of a number of instances of depreciation of property values and the difficulty attending the sale of real estate just now, even at greatly reduced figures, attention was called to the effect of the agitation of the question of labor arrayed against capital. It was thought by these very capable business men—men owning real estate and manufacturing interests—that much harm is done by the thoughtless manner in which newspapers are apt to discuss the labor question, many of them inciting the laboring classes to the perpetration of outrages against their employers. There is a great responsibility for good or evil in the press, in discussing themes in which the laboring classes are interested. Stirring up prejudices and jealousies, in the ranks of the laboring people, against the capitalist, may tickle the first, thereby increasing the sale of the paper, but in the end does irreparable injury to the city in which the business is conducted. It was even intimated that the recent disastrous fire, in which Elmira's principal manufactory was totally destroyed, disemploying hundreds of dependent workmen, was kindled by vicious men, instigated by such influences referred to above.

We cannot be too careful in discussing these measures in the presence of uneducated men. Newspapers should use great caution in attempting to array the working man against his employer. Great damage has already resulted to both from this sort of warfare, and more serious consequences remain to follow.

---

THE AMERICAN SOCIETY OF MICROSCOPISTS.—Never before was the usefulness of a convention of men, engaged in a common pursuit, more thoroughly demonstrated than in the recent meeting of this national society, in our city. In whatever occupation men may be employed, either for profit or pleasure, good will come of a co-mingling of them, in one place, to chat over their hobby—let it be what it may. The social feature of such gatherings of men is an immense benefit to them, permitting an acquaintanceship through the cultivation of which they are bettered and made really more valuable members of the community in which they reside. Take such a body of men as the microscopists of our country—cultured, educated, refined gentlemen, as a whole, the refining influence with which they are unconsciously surrounded can but elevate and ennoble those not so favorably endowed, and with whom they may be thrown in contact. Then, their influence in stimulating a desire for knowledge, for investigation, for science, is a great lever for good. For these reasons, among others, Elmira received full value in return for her generous hospitality to these scientific men who recently favored us with their presence. We feel more than repaid for the outlay in money, time and patience necessary for the entertainment of so large a company. They were delightful guests to receive into our families, and their departure was a matter for regret with those fortunate enough to secure their company.

As to the scientific value of the meeting, it was the most interesting in papers and discussions yet held by the society; replete with knowledge imparted one to another in discussing methods for microscopical investigation, as revealed in different appliances and the manner of using them; in preparing, mounting and staining objects for the microscope; in opinions of the structure and uses of minute forms of animated nature; in histological facts, bearing upon the evolution of our own species; these, and many other themes, were discussed with an ability and absorbing interest refreshing to the anxious investigator after knowledge. Then, the ingenuity of men in adapting complicated and exquisitely wrought instruments (worked out with a mathematical precision, according to unvarying laws of science) to the work of demonstrating certain scientific theories or facts, is something marvelous to contemplate, leading one irresistably to the conclusion that among scientific men, none are better versed in general science and mechanics than the successful microscopist. It requires a peculiar talent, too, an inexhaustible stock of patience, indomitable perseverance and persistence to constitute a successful investigator in microscopy. Hence, the pithy, logical and convincing arguments that these men bring to bear in proving a theory or discovery brought before their fellows. Really, the largest amount of information gleaned from this convention of scientists, came from their scholarly, terse, and genial discussions, which, had they been stenographically reported, would have formed a volume of great value to those interested in this favorite study. A sufficient evidence of the captivating qualities of microscopic study was abundantly portrayed in the very large body of gentlemen that were enticed hither, from all parts of the United States, at great cost and hindrance to personal business pursuits, to spend a week in imparting and obtaining knowledge bearing upon this fascinating science.

Again, we say the meeting was a thoroughly enjoyable and useful one to the microscopists of our country, foreshadowing increased interest in their science and a host of new and fascinated followers.

---

STRUCTURE OF THE BLOOD CORPUSCLE.—No scientific investigation, of recent date, has absorbed more time and patient study, to say nothing of the spirited controversy indulged in by the votaries of two different theories, than the question as to the structure of the human blood corpuscle. To the casual reader, the consideration of a purely scientific theme of this character, offers but little, if any, interest. But, that our readers may know what is going on in the scientific world, and at the same time have an inkling as to the methods pursued in the investigation of so minute a structure, and what is to be gained thereby, we will try to give a brief outline of what has been done in the way of scrutinizing our blood, detailing the theories constructed therefrom.

A universally recognized competent histologist,* a few years ago, proclaimed that he had discovered that the red blood corpuscle was a living structure, capable of movements in its interior, by means of a net-work (called a *reticulum*), that pervaded the corpuscle in every direction, holding within its meshes the *protoplasm* or lifeless substance of the corpuscle. This net-work, named *bioplasm*—or living matter—was said to be capable of great contractile and expansive power, by means of which certain transformations are produced in the interior of the corpuscle. When this insinuating and pervading *reticulum* contracts, granules are formed, because of the structure being forced into knots. In a state of relaxation of the membrane, or when widely expanded, the granules disappear. Treating the corpuscles with certain chemical re-agents (like bichromate of potash) the membrane is caused to contract quickly and firmly, so that the resulting granules and outstretching *reticulum* leading therefrom, are the more readily seen.

The importance attached to this theory is, that it is an ingenious argument to prove that the blood corpuscle is living matter (like the *amœba*, one of the lowest forms of animated nature), and that it is from the multiplication and "differentiation" of them that the entire human economy is constructed;—the blood corpuscle being one of the units which enter into the structure of the body.

This theory and "discovery" of the Histologist set a host of investigators at work to see the "reticulated structure" referred to. Then, commenced a bombardment of this structure that knocked the "bioplasson theory" into a complete ruin.

Dr. Lester Curtis, of Chicago, was, we believe, the first to attack the reticulated structure, in print, having brought to bear upon it the more modern and superior objectives of this day, until the meeting of the American Society of Microscopists, in August, demolished whatever remained of it.

In the meeting referred to, it was shown that what was thought to be a net-work, was really shadows cast from the imperfect adjustment of the objectives used. The granules were shown to be produced by the chemical action of the re-agents used, and were due to a shrinking of the protoplasm into forms of that character. And so disappeared the "bioplasson" or "reticulated structure" theory of the blood corpuscle.

Nevertheless, that the red blood corpuscle does *not* contain "living matter,"—*bioplasm*, is by no means disproved by the arguments and experiments adduced. It is quite in unison with the laws that govern the formation of the *rhizopods* and other primative and low orders of animals; so that, we should be prepared to receive the theory as possibly truthful and consistent, until a more successful investigator establishes, beyond the possibility of successful contradiction, its existence.

* Dr. Heitzman, of New York.

